# Predictive and prognostic value of preoperative serum tumor markers in resectable adenosqamous lung carcinoma

**DOI:** 10.18632/oncotarget.11703

**Published:** 2016-08-30

**Authors:** Qiongjie Zhi, Yuqian Wang, Xinyue Wang, Dongsheng Yue, Kai Li, Richeng Jiang

**Affiliations:** ^1^ Tianjin Medical University Cancer Institute & Hospital, National Clinical Research Center for Cancer, Tianjin, PR China; ^2^ Key Laboratory of Cancer Prevention and Therapy, Tianjin, PR China; ^3^ Department of Thoracic Oncology, Tianjin Lung Cancer Center, Tianjin Cancer Institute & Hospital, Tianjin, PR China; ^4^ Department of Lung Cancer, Tianjin Lung Cancer Center, Tianjin Cancer Institute & Hospital, Tianjin Medical University, Tianjin, PR China

**Keywords:** tumor marker, TMI, adenosquamous lung carcinoma, prognostic factor, EGFR mutation

## Abstract

**Background:**

Adenosquamous carcinoma is a rare and aggressive form of lung cancer. The prognostic and predictive value of preoperative serum tumor markers and frequency of EGFR mutations in adenosquamous lung carcinoma are unclear.

**Methods:**

We retrospectively analyzed data and samples collected from 106 radically resected adenosquamous lung carcinoma patients with pathological stage I-IIIA between 2008 and 2013. Correlations between serum tumor marker levels and EGFR mutations as well as survival parameters were analyzed and prognostic factors were identified.

**Results:**

Of the 106 adenosquamous lung carcinoma patients, 29 (27.4%) harbored EGFR mutations. By univariate analysis, advanced clinical stage (*P* = 0.009 for disease-free survival [DFS]; *P* = 0.046 for overall survival [OS]), larger tumor size (*P* = 0.001 for DFS; *P* = 0.002 for OS), regional lymph node metastasis (*P* = 0.024 for DFS; *P* = 0.030 for OS), higher NSE level (*P* = 0.002 for DFS; *P* < 0.001 for OS), and higher TMI (tumor marker index) (*P* = 0.009 for OS) were significantly correlated with a worse prognosis. By multivariate analysis, NSE (*P* = 0.014) was confirmed as independent predictor for DFS, while NSE (*P* = 0.001) and TMI (*P* = 0.038) were independent prognostic factors for OS.

**Conclusion:**

Adenosquamous lung carcinoma is an aggressive malignancy with relatively high EGFR mutation frequency. Elevated preoperative NSE level and TMI are adverse predictive and prognostic indicators.

## INTRODUCTION

Lung cancer is the leading cause of cancer-related deaths worldwide. Non-small cell lung cancer, predominantly includes adenocarcinoma, squamous cell carcinoma and large cell carcinoma, accounts for about 80% of lung cancers. Adenosquamous lung carcinoma is a relatively rare subtype of non-small cell lung cancer comprising of 0.3% to 5% of all non-small cell lung cancers [1, 2].

According to criteria of World Health Organization, adenosquamous lung carcinoma is a carcinoma displaying components of both squamous cell carcinoma and adenocarcinoma, with each component comprising at least 10% of the tumor [3]. Adenosquamous lung carcinoma is regarded as more aggressive and carries a worse prognosis compared to adenocarcinoma and squamous cell carcinoma [1, 4]. However, its biological behaviors based on clinicopathological factors are not well understood. Most adenosquamous lung carcinoma patients eventually develop local recurrence and/or distant metastasis even after complete tumor resection, and the overall survival rates remain low. Standard platinum-based doublet chemotherapy of advanced non-small cell lung cancer has limited efficacy, thus new therapies are needed.

Recent advancements in EGFR mutation targeted therapy led to a major paradigm shift in the treatment of non-small cell lung cancer. EGFR-sensitizing mutations are strongly associated with robust responses to EGFR tyrosine kinase inhibitors (EGFR-TKI) and improved progression-free survival (PFS). However, EGFR mutations are most common in Asian patients, nonsmokers, females and those with adenocarcinoma histology [5]. In squamous cell carcinoma, the EGFR mutation rate is reported to be approximately 5% [6]. Although several small studies have indicated that the frequency of EGFR mutation in adenosquamous lung carcinoma ranges from 15% to 44% in the East Asian population, the exact prevalence of EGFR mutation in adenosquamous lung carcinoma is still not clear.

In recent years, serum tumor markers, including neuron-specific enolase (NSE) [7-9], carcinoembryonic antigen (CEA) [10, 11], cytokeratin-19 fragments (Cyfra21-1) [12] and squamous cell carcinoma antigen (SCCA) [13] have been extensively investigated, and considered potentially predictive and prognostic in non-small cell lung cancer. However, little is known about the predictive and prognostic value of the tumor markers in patients with adenosquamous lung carcinoma. Here, we investigated the clinicopathological characteristics of adenosquamous lung carcinoma patients who underwent surgery and explored the predictive and prognostic value of serum tumor markers NSE, CEA, Cyfra21-1 and SCCA.

## RESULTS

### Patient characteristics

The clinical characteristics of the 106 adenosquamous lung carcinoma patients are shown in Table 1. Sixty-three patients (59.4%) were men and 42 (39.6%) were never-smokers, with an age range of 35 to 83 years (median, 60 years). Among the patient histology, 49 (46.3%) were adenocarcinoma predominant, 26 (24.5%) squamous cell carcinoma predominant, and 31 (29.2%) with equal adenocarcinoma and squamous cell carcinoma. The distribution of clinical stages was as follows: 41 stage I, 19 stage II, and 46 stage IIIA. Eight patients (7.5%) received pneumonectomy, 89 (84.0%) received lobectomy, and 9 (8.5%) underwent wedge resection. EGFR mutations were detected in 27.4% (29/106) of 106 patients. Among the 29 patients with mutations, 13 harbored exon 19 deletions (del19), 13 had point mutations in exon 21 (12 were L858R, and one was L861Q), two had G719X in exon 18, and one possessed dual mutations of G719X in exon 18 and S768I in exon 20. No T790M mutations were detected in these patients.

Of the 106 adenosquamous lung carcinoma patients, 63 (59.5%) were treated with platinum-based adjuvant chemotherapy, 10 (9.4%) underwent platinum-based adjuvant chemoradiotherapy, and 68 (64.2%) had recurrent disease during the study follow-up period. The median DFS was 15.0 months (95% confidence interval [CI]: 9.5-20.4). In addition, 43 (40.6%) recurrent patients received systemic chemotherapy, 28 (26.4%) patients also received radiotherapy as local therapy, and only 5 (4.7%) received EGFR-TKIs. At the end of the last follow up, 69 (65.1%) patients had died. Median overall survival was 26.0 months. The survival rates of 1, 2 and 5-year were 76.4%, 52.9%, and 25.3%, respectively. Among the 68 patients with recurrent disease, the initially recurrent sites were the loco-regional or lung (33.8%, 23/68), bone (10.3%, 7/68), brain (16.2%, 11/68) and liver (7.3%, 5/68), 22 (32.4%) patients had multiple recurrent sites.

**Table 1:** Patient characteristics

Variable	No. of Patients	%
Age (years)		
Median (range)	60 (35-83)	
< 60	50	47.2
≥ 60	56	52.8
Gender		
Male	63	59.4
Female	43	40.6
Smoking history		
Never	42	39.6
Ever	64	60.4
Component		
Adenocarcinoma predominant	49	46.3
Squamous cell carcinoma predominant	26	24.5
Equal proportion	31	29.2
Clinical stage		
I	41	38.7
II	19	17.9
IIIA	46	43.4
Tumor size		
≤ 3 cm	36	44.0
> 3 cm	70	56.0
Regional lymph node metastasis		
No	47	44.3
Yes	59	55.7
Operative approaches		
Pneumonectomy	8	7.5
Lobectomy	89	84.0
Wedge resection	9	8.5
Adjuvant treatment		
Chemotherapy	63	59.5
Chemoradiotherapy	10	9.4
Others/none	33	31.1
EGFR mutation		
Wild-type	77	72.6
Exon 19 deletion	13	12.3
L858R substitution	12	11.3
Others	4	3.8
NSE		
≤ 15.2 ng/ml	68	64.2
> 15.2 ng/ml	38	35.8
CEA		
≤ 5.0 ng/ml	56	52.8
> 5.0 ng/ml	50	47.2
Cyfra21-1		
≤ 3.3 ng/ml	48	45.3
> 3.3 ng/ml	58	54.7
SCCA		
≤ 1.5 ng/ml	90	84.9
> 1.5 ng/ml	16	15.1
Tumor marker index (TMI)		
≤ 0.54	17	16.0
> 0.54	89	84.0
Recurrent sites (n = 68)		
Loco-regional or lung	23	33.8
Bone	7	10.3
Brain	11	16.2
Liver	5	7.3
Multiple	22	32.4
Post-recurrence treatment Systemic chemotherapy		
No	63	50.4
Yes	43	40.6
Radiotherapy		
No	78	73.6
Yes	28	26.4
EGFR-TKI		
No	101	95.3
Yes	5	4.7
Total	106	100

### Association of serum tumor marker levels with clinicopathological characteristics

As shown in Table 2, preoperative NSE, CEA, Cyfra21-1 and SCCA levels were elevated in 35.8%, 47.2%, 54.7% and 15.1% of adenosquamous lung carcinoma patients, respectively. Elevated preoperative NSE levels were closely associated with advanced clinical stage (*P* = 0.006), smoking history (*P* = 0.036) and regional lymph node metastasis (*P* = 0.005). In addition, median NSE levels in patients with larger tumor size were higher than those with smaller tumor size (14.7 *versus* 13.7 ng/ml, *P* = 0.045). And increased SCCA levels were found to be correlated with tumor size (*P* = 0.011). Neither CEA nor Cyfra21-1 levels were correlated with any clinical parameter in adenosquamous lung carcinoma patients. Median levels and positive rates for NSE, CEA, Cyfra21-1 or SCCA were similar regardless of EGFR mutation status in adenosquamous lung carcinoma patients. Similarly, no differences were found in positive rates and median levels of these tumor markers between del19 and L858R subtypes.

Moreover, as shown in Supplementary Table 1, EGFR mutations were found more frequently in women (48.8% *versus* 12.7%, χ^2^ = 16.795, *P* < 0.001), never-smokers (42.9% *versus* 17.2%, χ^2^ = 8.408, *P* = 0.004) and younger patients (36.0% *versus* 19.6%, χ^2^ = 3.556, *P* = 0.059).

**Table 2:** The association between serum tumor markers and the clinicopathological characteristics

Variable	NSE median (IQR)	CEA median (IQR)	Cyfra21-1 median (IQR)	SCCA median (IQR)	NSE > 15.2 ng/ml (%)	CEA > 5.0 ng/ml (%)	Cyfra21-1 > 3.3 ng/ml (%)	SCCA > 1.5 ng/ml (%)
Component								
Adenocarcinoma predominant (n = 49)	14.2 (11.6-18.3)	5.5 (3.0-16.1)	3.8 (2.4-5.1)	0.8 (0.4-1.3)	18 (36.7)	26 (53.0)	27 (55.1)	7 (14.3)
Squamous cell carcinoma predominant (n = 26)	14.7 (12.8-20.6)	5.7 (3.2-13.9)	3.5 (2.5-5.3)	0.8 (0.5-1.0)	11 (42.3)	15 (57.7)	17 (65.4)	3 (11.5)
Equal proportion (n = 31)	13.8 (10.5-16.2)	3.3 (2.2-7.0)	3.0 (2.1-5.2)	0.7 (0.3-1.3)	9 (29.0)	9 (29.0)	14 (45.2)	6 (19.4)
*P*-value	0.079	0.169	0.568	0.987	0.573	0.052	0.310	0.735
Clinical stage								
I (n = 41)	13.5 (11.4-13.5)	3.6 (2.2-11.6)	3.2 (2.1-4.6)	0.8 (0.3-1.2)	7 (17.1)	16 (39.0)	20 (48.8)	3 (7.3)
II ( n = 19)	15.1 (11.6-19.0)	5.3 (3.0-8.8)	3.5 (2.5-5.2)	1.0 (0.5-1.8)	9 (47.4)	10 (52.6)	11 (57.9)	5 (26.3)
IIIA ( n = 46)	14.9 (12.1-18.6)	5.2 (3.0-21.9)	4.0 (2.4-5.3)	0.7 (0.4-1.2)	22 (47.8)	24 (52.2)	27 (58.7)	8 (17.4)
*P*-value	0.016	0.484	0.422	0.642	0.006	0.517	0.620	0.205
Tumor size								
≤ 3 cm (n = 36)	13.7 (11.6-15.6)	3.3 (2.0-9.6)	3.3 (1.9-4.2)	0.6 (0.4-1.0)	10 (27.8)	14 (38.9)	18 (50.0)	1 (2.7)
> 3 cm (n = 70)	14.7 (12.1-18.5)	5.2 (3.0-15.6)	3.9 (2.5-5.4)	0.9 (0.4-1.3)	28 (40.0)	36 (51.4)	40 (57.1)	15 (21.4)
*P*-value	0.045	0.448	0.241	0.207	0.214	0.221	0.484	0.011
Regional lymph node metastasis								
No (n = 59)	13.7 (11.6-15.0)	3.2 (2.2-12.3)	3.2 (2.0-5.2)	0.9 (0.3-1.4)	28 (47.5)	32 (54.2)	35 (59.3)	10 (16.9)
Yes (n = 47)	15.1 (12.0-19.0)	5.3 (3.2-16.3)	3.8 (2.5-5.2)	0.7 (0.4-1.2)	10 (21.3)	18 (38.3)	23 (48.9)	6 (12.8)
*P*-value	0.092	0.197	0.370	0.534	0.005	0.102	0.293	0.550
EGFR mutation								
Wild-type (n = 77)	14.3 (12.4-18.3)	4.1 (2.7-12.4)	3.7 (2.5-5.3)	0.9 (0.4-1.3)	29 (37.7)	33 (42.9)	45 (58.4)	14 (19.2)
Exon 19 deletion (n = 13)	12.3 (10.6-16.6)	5.2 (3.2-23.4)	2.5 (1.8-3.9)	0.6 (0.5-0.8)	4 (30.8)	7 (53.8)	6 (46.2)	0 (0.0)
L858R substitution (n = 12)	14.4 (12.2-25.0)	6.9 (2.2-16.3)	2.8 (1.6-5.4)	0.6 (0.4-1.0)	4 (33.3)	7 (58.3)	5 (41.7)	1 (9.1)
Others (n = 4)	14.6 (10.5-20.0)	5.9 (3.6-8.3)	3.6 (2.8-4.2)	1.8 (0.4-2.3)	1 (25.0)	3 (75.0)	2 (50.0)	1 (25.0)
*P*-value	0.679	0.314	0.801	0.062	0.977	0.477	0.621	0.277
Total (n = 106)	14.3 (11.9-18.3)	4.6 (2.7-13.0)	3.5 (2.4-5.2)	0.8 (0.4-1.25)	38 (35.8)	50 (47.2)	58 (54.7)	16 (15.1)

### Association of serum tumor markers, TMI and EGFR mutation status with DFS and OS

**Figure 1:**
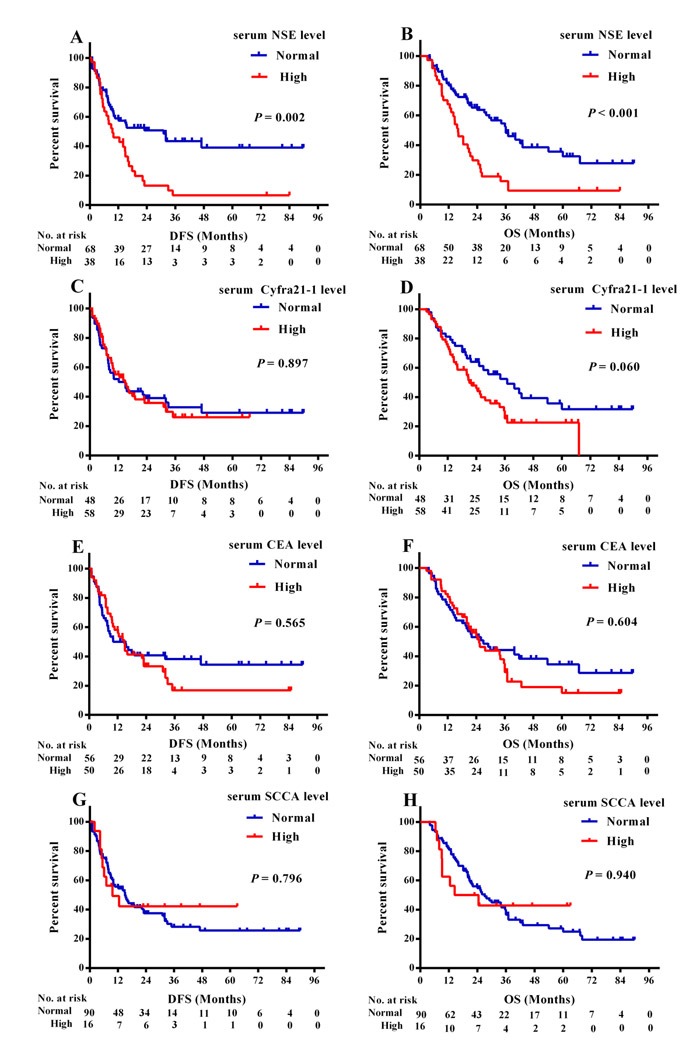
Kaplan-Meier survival curves of DFS and OS based on different levels of serum tumor markers **A.** DFS and **B.** OS based on different levels of NSE; **C.** DFS and **D.** OS based on different levels of Cyfra21-1; **E.** DFS and **F.** OS based on different levels of CEA; **G.** DFS and **H.** OS based on different levels of SCCA;

Among the 106 adenosquamous lung carcinoma patients, 38 had elevated NSE levels, 50 elevated CEA, 58 elevated Cyfra21-1 and 16 elevated SCCA. DFS and OS were significantly shorter in patients with elevated NSE (9.6 *versus* 20.5 months, log-rank χ^2^ = 9.638, *P* = 0.002 for DFS, Figure 1A; 16.0 *versus* 36.0 months, log-rank χ^2^ = 15.330, *P* < 0.001 for OS, Figure 1B). Patients with elevated Cyfra21-1 exhibited similar DFS (14.8 *versus* 15.0 months, log-rank χ^2^ = 0.017, *P* = 0.897, Figure 1C) but shorter OS (22.0 *versus* 37.0 months, log-rank χ^2^ =3.533, *P* = 0.060, Figure 1D). Neither CEA nor SCCA was correlated with any effect on DFS or OS (CEA: *P* = 0.565 for DFS, Figure 1E; *P* = 0.604 for OS, Figure 1F; SCCA: *P* = 0.796 for DFS, Figure 1G; *P* = 0.940 for OS, Figure 1H).

**Figure 2:**
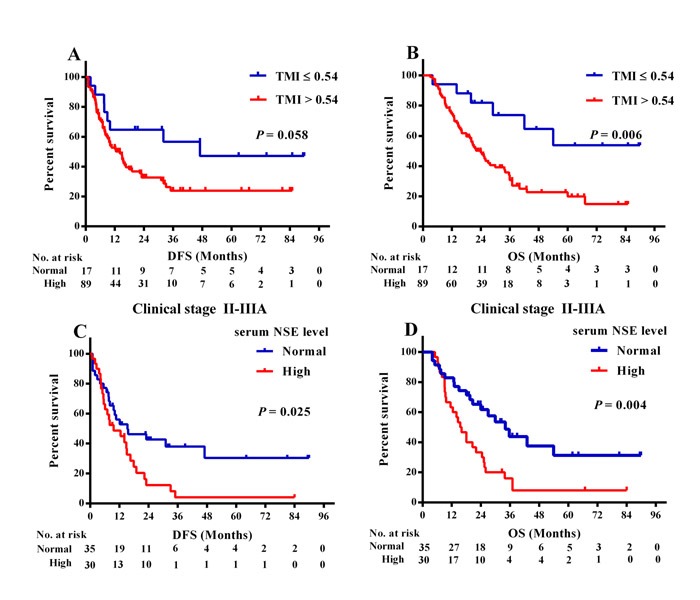
Kaplan-Meier survival curves of DFS and OS based on TMI as well as NSE levels **A.** DFS and **B.** OS based on different levels of TMI. **C.** DFS and **D.** OS based on different NSE levels in stage II-IIIA patients (*n* = 65).

The relationship between the TMI and survival in adenosquamous lung carcinoma patients is shown in Figure 2. There were 17 patients with TMI ≤ 0.54 and 89 patients with TMI > 0.54. The OS of patients with a TMI ≤ 0.54 was longer than patients with a TMI > 0.54 but no difference in DFS was found between the two groups (47.0 *versus* 14.0 months, log-rank χ^2^ = 3.600, *P* = 0.058 for DFS, Figure 2A; Not Reached [NR] *versus* 24.0 months, log-rank χ^2^ = 7.534, *P* = 0.006 for OS, Figure 2B).

Moreover, in those patients with stage II and IIIA diseases, elevated NSE levels were associated with shorter DFS and OS (9.6 *versus* 15.3 months, log-rank χ^2^ = 5.036, *P* = 0.025 for DFS, Figure 2C; 15.5 *versus* 34.0 months, log-rank χ^2^ = 8.479, *P* = 0.004 for OS, Figure 2D), while this relationship between increased NSE and DFS or OS was not found in stage I patients.

As shown in Table 3, similar DFS and OS were observed in patients regardless of EGFR mutation status (*P* = 0.893 for DFS; *P* = 0.642 for OS). Of the 29 adenosquamous lung carcinoma patients with EGFR mutations, no difference was found in DFS and OS between del19 and L858R subgroups (*P* = 0.595 for DFS; *P* = 0.778 for OS).

**Table 3:** Univariate analysis of DFS and OS

	DFS			OS		
Variable	Median (months)	HR (95%CI)	*P*-value	Median (months)	HR (95%CI)	*P*-value
Total (n = 106) Age (years)						
< 60	12.1	Reference	0.492	34.1	Reference	0.159
≥ 60	16.5	0.846 (0.526-1.362)		22.0	1.411 (0.874-2.280)	
Gender						
Male	14.0	Reference	0.237	24.5	Reference	0.619
Female	15.5	1.350 (0.821-2.221)		29.0	1.132 (0.695-1.844)	
Smoking history						
Never	15.0	Reference	0.461	27.8	Reference	0.986
Ever	15.0	1.206 (0.733-1.986)		38.7	0.996 (0.610-1.624)	
Component						
Adenocarcinoma predominant	12.1	Reference	0.645	24.5	Reference	0.780
Squamous cell carcinoma predominant	19.0	0.777 (0.429-1.408)		34.0	0.910 (0.507-1.635)	
Equal proportion	15.3	0.819 (0.462-1.453)		33.8	0.816 (0.463-1.440)	
Clinical stage						
I	32.0	Reference	0.009	42.0	Reference	0.046
II	15.5	1.447 (0.706-2.966)		19.5	1.652 (0.815-3.550)	
IIIA	9.6	2.335 (1.345-4.052)		24.0	1.963 (1.148-3.357)	
Tumor size						
≤ 3 cm	47.0	Reference	0.001	43.0	Reference	0.002
> 3 cm	9.9	2.733 (1.532-4.877)		20.5	2.434 (1.403-4.225)	
Regional lymph node metastasis						
No	32.0	Reference	0.024	37.0	Reference	0.030
Yes	12.1	1.775 (1.080-2.918)		22.0	1.720 (1.055-2.804)	
Operative approaches						
Pneumonectomy	12.5	Reference	0.203	16.0	Reference	0.167
Lobectomy	15.5	0.511 (0.242-1.078)		26.0	0.552 (0.247-1.103)	
Wedge resection	10.0	0.631 (0.218-1.802)		29.0	0.778 (0.291-2.081)	
Adjuvant treatment						
Chemotherapy	14.8	Reference	0.538	25.2	Reference	0.720
Chemoradiotherapy	9.6	1.372 (0.667-2.823)		25.5	0.782 (0.333-1.836)	
Others/none	16.5	0.867 (0.493-1.524)		36.0	0.830 (0.484-1.423)	
EGFR mutation						
Wild-type	15.3	Reference	0.893	33.8	Reference	0.642
Mutant	14.0	0.963 (0.560-1.656)		25.5	0.877 (0.504-1.527)	
NSE						
≤ 15.2 ng/ml	20.5	Reference	0.002	36.0	Reference	< 0.001
> 15.2 ng/ml	9.6	2.103 (1.300-3.401)		16.0	2.504 (1.555-4.031)	
CEA						
≤ 5.0 ng/ml	15.3	Reference	0.566	25.9	Reference	0.605
> 5.0 ng/ml	14.8	1.150 (0.713-1.856)		25.4	1.133 (0.706-1.819)	
Cyfra21-1						
≤ 3.3 ng/ml	15.0	Reference	0.897	37.0	Reference	0.063
> 3.3 ng/ml	14.8	1.032 (0.639-1.666)		22.0	1.593 (0.975-2.601)	
SCCA						
≤ 1.5 ng/ml	15.0	Reference	0.723	27.0	Reference	0.986
> 1.5 ng/ml	9.6	0.881 (0.436-1.780)		14.5	0.994 (0.491-2.011)	
Tumor marker index (TMI)						
≤ 0.54	47.0	Reference	0.063	NR	Reference	0.009
> 0.54	14.0	2.020 (0.961-4.246)		24.0	3.071 (1.320-7.142)	
Clinical stage I (n = 41) NSE						
≤ 15.2 ng/ml (n = 33)	32.0	Reference	0.147	42.0	Reference	0.132
> 15.2 ng/ml (n = 8)	9.0	2.017 (0.781-5.209)		15.2	2.027 (0.809-5.081)	
TMI						
≤ 0.54 (n = 11)	NR	Reference	0.213	NR	Reference	0.113
> 0.54 (n = 30)	15.0	1.998 (0.671-5.949)		36.0	2.399 (0.812-7.090)	
Clinical stage II-IIIA (n = 65) NSE						
≤ 15.2 ng/ml (n = 35)	15.3	Reference	0.028	34.0	Reference	0.005
> 15.2 ng/ml (n = 30)	9.6	1.926 (1.075-3.453)		15.5	2.345 (1.296-4.243)	
TMI						
≤ 0.54 (n = 6)	7.6	Reference	0.360	NR	Reference	0.092
> 0.54 (n = 59)	12.5	1.632 (0.571-4.663)		20.7	3.409 (0.817-14.217)	

### Univariate and multivariate analysis of prognostic factors

By univariate analysis, advanced clinical stage (*P* = 0.009 for DFS; *P* = 0.046 for OS), larger tumor size (*P* = 0.001 for DFS; *P* = 0.002 for OS), regional lymph node metastasis (*P* = 0.024 for DFS; *P* = 0.030 for OS), higher NSE level (*P* = 0.002 for DFS; *P* < 0.001 for OS), and higher TMI (*P* = 0.009 for OS) were significantly correlated with a worse prognosis (Table 3).

By multivariate analysis, elevated NSE (hazard ratio [HR] = 1.862; 95% CI: 1.131-3.066; *P* = 0.014) was confirmed as independent predictors for DFS, while increased NSE (HR = 2.199; 95% CI: 1.355-3.568; *P* = 0.001) and TMI (HR = 2.479; 95% CI: 1.050-5.855, *P* = 0.038) were independent prognostic factors for OS (Table 4).

**Table 4:** Multivariate analysis of DFS and OS

		DFS			OS	
Variable	HR	95%CI	*P*-value	HR	95%CI	*P*-value
Clinical stage (I vs. II-IIIA)	1.589	0.922-2.738	0.096			
NSE (> 15.2 ng/ml vs. ≤ 15.2 ng/ml )	1.862	1.131-3.066	0.014	2.199	1.355-3.568	0.001
Tumor marker index (TMI) (> 0.54 vs. ≤ 0.54)				2.479	1.050-5.855	0.038

## DISCUSSION

Preoperative serum tumor markers secreted by tumor cells might reflect intratumor heterogeneity, and have been investigated as tools for predicting disease progression, either alone, or in combination with clinical variables [14]. However, the predictive and prognostic significance of the tumor markers for adenosquamous lung carcinoma remains largely unknown. EGFR mutations are most common in adenocarcinoma, while their frequency in the context of adenosquamous lung carcinoma remains controversial.

In this study, we found that the EGFR mutation rate (29/106, 27.4%) was relatively high in adenosquamous lung carcinoma patients but had no effect on disease-free survival or overall survival. Furthermore, we demonstrated that elevated NSE and TMI were independently unfavorable predictors. No significant difference in disease-free survival or overall survival were observed between patients with high carcinoembryonic antigen (CEA), cytokeratin 19 fragments (Cyfra21-1) or squamous cell carcinoma antigen (SCCA) levels compared to those with lower levels.

Several studies have demonstrated that the survival rate of adenosquamous lung carcinoma patients is shorter than other types of non-small cell lung cancer. The reported 5-year survival rate for adenosquamous lung carcinoma patients has varied between 6.2% and 25.4% [1, 15-18]. In the present study, we observed that the 5-year survival rate to be approximately 25.3%. In addition, the high frequency of lymph nodal metastases (59/106, 55.7%) and advanced pathological stage (46/106, 43.4%) at surgery underscores the aggressive behavior of adenosquamous lung carcinoma. Moreover, more than half adenosquamous lung carcinoma patients who underwent complete surgical resection experienced distant metastases or local recurrences (68/106, 64.2%) including distant brain metastases (11/68), consistent with the results of a prior study [1].

A previous study found that the amount of adenocarcinoma components did not affect the survival rate, while another reported that adenocarcinoma predominant tumors were considered to be a worse prognostic factor [18-20]. Conversely, Gawrychowski et al observed that a balance of squamous and adenocarcinoma components had better prognosis than either being predominant [17]. In the present study we found no significant difference in disease-free survival or overall survival between adenosquamous lung carcinoma patients based on dominant component. This difference in results between our study and others could be attributed to different sample sizes, or the fact that biopsies come from small regions of the tumor which may underrepresent adenosquamous carcinoma histologic characteristics.

EGFR mutation has been reported to be a good outcome predictor for patients with non-small cell lung cancer. Consistent with previous studies [21, 22], we observed that EGFR mutation frequency was increased in never-smokers and in females. However, no correlation between EGFR mutation status and patient survival was observed, which may result from the low incidence of EGFR-TKI treatment in this cohort (5/29). Two additional reports were unable to detect a significant association between EGFR mutation and prognosis in patients who did not receive EGFR-TKI therapy [23, 24]. In addition, a prior study showed an adenosquamous lung carcinoma patient harboring EGFR-sensitizing mutation had a remarkable response to gefitinib [25]. Therefore EGFR tyrosine kinase inhibitors would be a reasonable therapeutic option to adenosquamous lung carcinoma patients due to the relatively high frequency of EGFR mutations in this cohort.

According to previous studies, serum NSE level in non-small cell lung cancer may reflect the heterogeneity and neuroendocrine phenotype and was as a prognostic factor for neuroendocrine lung tumors [26, 27]. However, the predictive value of NSE in non-small cell lung cancer are unclear, since several studies did not observe a prognostic role, while others have reported that elevated NSE is associated with poor prognosis [7, 8, 28]. NSCLC with neuroendocrine properties was previously reported to be chemosensitive but was associated with poorer outcomes similar to small cell lung cancer.[29] In another study, elevated serum NSE predicted NSCLC resistance to EGFR-TKIs and it was speculated that transition to small cell lung cancer occurred after acquisition of EGFR-TKI resistance [9]. Interestingly, sequential NSE level measurement has predicted tumor recurrence, dropping during effective treatment, but rising again after relapse [30, 31]. And Bastide et al. found adenosquamous lung carcinoma is more phenotypically similar to neuroendocrine tumors compared to adenocarcinoma lung cancer or squamous cell lung cancer [32]. Further, we confirmed that high NSE level in adenosquamous lung carcinoma confers a poorer prognosis. A strong positive association was identified between preoperative NSE and clinical stage as well as lymph node metastasis. Elevated NSE levels gave a poorer prognosis to patients with advanced staged tumors compared to those with earlier stage disease. These results suggest that NSE reflects tumor burden and aggressiveness, consistent with previous studies. Therefore, our results suggest that these patients should undertake a more aggressive clinical course.

Cyfra21-1 and CEA have been confirmed as valuable prognostic factors for NSCLC. Previous studies found that Cyfra21-1 tends to be more useful for squamous cell carcinoma diagnosis while CEA is predictive for adenocarcinoma.[10, 12, 33] In our study, elevated levels of neither Cyfra21-1 nor CEA were associated adenosquamous lung carcinoma patient prognosis. Interestingly, the TMI evaluating both Cyfra21-1 and CEA simultaneously was an independent prognostic marker for overall survival (HR = 2.479, 95% CI: 1.050-5.855, *P* = 0.038). This result might reflect that adenosquamous lung carcinoma stems from a monoclonal expansion of a single mutant progenitor cell clone, maintaining both adenocarcinoma and squamous cell carcinoma characteristics [32, 34, 35]. However, the molecular mechanism of trans-differentiation into adenocarcinoma or squamous cell carcinoma requires furthermore exploration.

Our retrospective study has some limitations. First, some data was censored, the patient sample size was relatively small, and patients undertook different chemotherapeutic regimens. In addition, we did not investigate the adenosquamous lung carcinoma components separately so it is not known which harbored identified EGFR mutations. It has been reported that adenocarcinoma and squamous cell carcinoma components can possess the same EGFR mutation, suggesting that the histologic origin of adenosquamous lung carcinoma can be monoclonal [22].

In conclusion, our results demonstrate that elevated preoperative NSE level has adverse predictive and prognostic value. In addition, TMI based on serum CEA and Cyfra21-1 is also an independent prognostic for overall survival. A larger prospective study will be needed to confirm these results.

## PATIENTS AND METHODS

### Patients

A total of 106 adenosquamous lung carcinoma patients who underwent curative-intent complete resection between January 2008 and January 2013 at the Tianjin Medical University Cancer Institute & Hospital were enrolled in this retrospective study. The study was approved by the Institutional Review Board of Tianjin Medical University Cancer Institute & Hospital and informed consent was obtained from all patients. The following patients are excluded: (1) locally advanced (stage IIIB), metastasized (stage IV), or postsurgically relapsed adenosquamous lung carcinoma; (2) insufficient tissue specimens or unavailable for genetic analysis; (3) blood samples not obtained before operation, or serum tumor marker records were unavailable; (4) patients who died within one month after surgery; (5) preoperative chemotherapy or radiotherapy; and (6) history of second primary cancer diagnosed within 5 years. Patient characteristics are shown in Table 1.

In terms of World Health Organization histological classification (3rd edition), if the percentage of one of the tumor components was less than 10%, such cancer was defined according to predominating texture and added ‘with elements’ [3]. Such patients were not taken into account in this study. If each of the components account for 40-60%, such cases were regarded as balanced and classified according to adenocarcinoma and squamous cell carcinoma characteristics shown in each case into the following subgroups: Adenocarcinoma predominant group, in which the adenocarcinoma component was equal to or more than 60% of tumor cells; Squamous cell carcinoma predominant group, in which the adenocarcinoma component was less than 50%; and equal adenocarcinoma and squamous cell carcinoma group, in which the adenocarcinoma component was 50%-60%.

### Measurement of serum NSE, CEA, Cyfra21-1 and SCCA levels and EGFR mutations

Serum concentrations of NSE, CEA, Cyfra21-1 and SCCA were measured within 2 weeks before surgery by electrochemiluminescence immunoassay on Roche Analytics E170 Immunology Analyzer (Roche Diagnostics, China). Based on the manufacturer's recommendation, the following cut-offs for serum marker levels were used: NSE 15.2 ng/ml, CEA 5.0 ng/ml, Cyfra21-1 3.3 ng/ml and SCCA 1.5 ng/ml.

Tumor marker index (TMI) was defined by the geometric mean of normalized CEA and Cyfra21-1. It was calculated as described recently (TMI = square root of CEA concentration/5.0 ng/ml × Cyfra21-1 concentration/3.3 ng/ml). The cut-off point for TMI was 0.54 according to results of Muley et al [36].

EGFR mutations were identified by polymerase chain reaction based direct sequencing to detect the nucleotide sequencing of the kinase domain of EGFR of individual exons (18-21 exons).

### Statistical analysis

Data are presented as the mean ± SD or median and IQR (intraquartile range). Fisher's exact test or chi-square test was conducted to compare the distribution of categorical variables. The Mann-Whitney U-test or Kruskal-Wallis one-way ANOVA test was used to compare continuous data. Overall survival (OS) was defined as the time from the date of surgery to the date of final follow-up or death from any cause. Disease-free survival (DFS) was calculated from the date of surgery until the date of the first recurrence or death from any cause. The survival curves were estimated using the Kaplan-Meier method, and differences were evaluated using the log-rank test. Hazard ratios (HR) and 95% confidence intervals (95% CI) were assessed by univariate and multivariate analyses using the Cox proportional hazards model. The independent prognostic factor was estimated by the Cox proportional hazards model using stepwise regression (backward selection). A *P* value less than 0.05 was considered to indicate a statistically significant difference. SPSS 22.0 for Windows (SPSS, Chicago IL) was performed to analyze data.

## SUPPLEMENTARY MATERIALS TABLE


