# Supplementary material for: Predictive and prognostic value of preoperative serum tumor markers in resectable adenosqamous lung carcinoma
Source: Oncotarget. 2016 Aug 30;7(40):64798–809. doi: 10.18632/oncotarget.11703 (PMC5323117; doi:10.18632/oncotarget.11703)
Supplement: Supplementary file 1 [file oncotarget-07-64798-s001.pdf]

Predictive and prognostic value of preoperative serum tumor markers in resectable adenosqamous lung carcinoma

Supplementary Material

Supplementary Table 1: The association between EGFR mutation status and the clinicopathological characteristics

Variable	No. of Patients	EGFR wild-type (%)	EGFR mutation (%)	χ^2	*P*-value
**Age (years)**					
< 60	50	32 (64.0)	18 (36.0)	3.556	**0.059**
≥ 60	56	45 (80.4)	11 (19.6)		
**Gender**					
Male	63	55 (87.3)	8 (12.7)	16.795	**< 0.001**
Female	43	22 (51.2)	21 (48.8)		
**Smoking history**					
Never	42	24 (57.1)	18 (42.9)	8.408	**0.004**
Ever	64	53 (82.8)	11 (17.2)		
**Component**					
Adenocarcinoma predominant	49	33 (67.3)	16 (32.7)	1.693	0.429
Squamous cell carcinoma predominant	26	19 (73.1)	7 (26.9)		
Equal proportion	31	25 (80.6	6 (19.4)		
**Clinical stage**					
I	41	31 (75.6)	10 (24.3)	0.411	0.814
II	19	14 (73.7)	5 (26.3)		
IIIA	46	32 (69.6)	14 (30.4)		
**Tumor size**					
≤ 3 cm	36	24 (66.7)	12 (33.3)	0.979	0.322
> 3 cm	70	53 (75.7)	17 (24.3)		
**Regional lymph node metastasis**					
No	47	37 (78.7)	10 (21.3)	1.572	0.210
Yes	59	40 (67.8)	19 (32.2)		
**Total**	106	77 (72.6)	29 (27.4)		
